# Dental Considerations in Children with Glucose-6-phosphate Dehydrogenase Deficiency (Favism): A Review of the Literature and Case Report

**DOI:** 10.1155/2015/506459

**Published:** 2015-09-07

**Authors:** Daniela Hernández-Pérez, Claudia Butrón-Téllez Girón, Socorro Ruiz-Rodríguez, Arturo Garrocho-Rangel, Amaury Pozos-Guillén

**Affiliations:** Pediatric Dentistry Postgraduate Program, Faculty of Dentistry, San Luis Potosi University, 2 Dr. Manuel Nava, Zona Universitaria, 78290 San Luis Potosí, SLP, Mexico

## Abstract

Glucose-6-phosphate dehydrogenase (G6PD) deficiency is an uncommon inherited enzyme deficiency characterized by hemolytic anemia, caused by the inability of erythrocytes to detoxify oxidizing agents such as drugs, infectious diseases, or fava bean ingestion. In this later case, the disorder is known as favism. The aim of the present report was to present a review of the literature in this disease, to describe a case report concerning an affected 9-year-old male, and to review the main implications and precautions in pediatric dental management.

## 1. Introduction

Although not commonly seen, metabolic disorders can significantly impact on dental treatment in diverse ways, and its knowledge is essential for the safe management of pediatric patients, including special precautions to facilitate this purpose. For some highly sensitive children, consuming certain drugs or foods will elicit adverse reactions, which could imply a debilitating or even life-threatening experience for them [1, 2]. Glucose-6-phosphate dehydrogenase (G6PD) is a cytoplasmic X chromosome-linked enzyme that prevents oxidative damage to cells by promoting detoxification of free radicals. This metabolic pathway involves the production of nicotinamide adenine dinucleotide phosphate (NADPH), which participates in the glutathione cycle, thus protecting the cell against hydrogen peroxide-induced damage and ensuring an intracellularly balanced oxidative environment [3, 4]. G6PD is expressed in all tissues; however, when the enzyme is abnormally low or deficient, this biochemical process is markedly reduced in red blood cells, rendering these cells strongly vulnerable to oxidative stress [3]. Clinical manifestations of G6PD include acute or chronic hemolytic anemia with hemoglobin denaturation and puddling (a pathological condition known as* methahemoglobinemia*), neonatal hyperbilirubinemia, and neonatal jaundice, although the disease is clinically asymptomatic and rarely fatal [1, 5, 6]. In children with G6PD deficiency, hemolysis can be precipitated by diverse factors, such as oxidative drugs, infectious diseases, and ingesting fava beans [7, 8].

G6PD deficiency is considered as the most common human genetic disorder worldwide, with estimated >400 million people carrying a mutation that causes this enzyme deficiency, mainly throughout tropical and subtropical regions. Its prevalence is associated with the geographic distribution of* Plasmodium falciparum* malaria infection, which has led to the theory that carriers of G6PD deficiency are partially protected against this infection, the deadliest form of malaria [3]. Highest prevalence has been reported in Africa, Mediterranean Europe, the Middle East, Southeast Asia, and the Pacific Islands [3, 9]. In the USA, black males are most commonly affected, with a prevalence of around 10% [6]. The high prevalence of G6PD has been reported in studies and surveys from Brazil, Ecuador, Jamaica, Colombia, and Cuba, whereas low prevalence rates (<0.05%) have been documented in Argentina, Bolivia, Mexico, Peru, and Uruguay [4, 12].

The association between the G6PD deficiency and ingestion of fava beans, even inhalation of the plant's pollen, has been recognized for centuries, and it has been mentioned that “Pythagoras forbade his followers from eating fava beans, possibly because of their pathological effects” [11]. During the second half of the twentieth century, severe hemolytic anemia in individuals ingesting fava beans, more commonly in children, was reported [11]. Fava beans are presumed to precipitate oxidative damage by means of an unknown component, possibly vicine, convicine, or isouramil, all of which are glucosides found in the digestive tract that are capable of causing hemolysis in G6PD-deficient children [12, 13].

The aim of this report was to conduct an exhaustive search of the medical and dental literature in order to locate, appraise, and synthesize the best available evidence associated with G6PD in the pediatric dentistry field. In addition, our aim was to report a case of a child with the disease and to discuss the main considerations and precautions to take into account during treatment at the dental office. The report and publication of the present case were carried out in accordance with international ethical norms and authorized by both of the patient's parents and the Research Ethics Committee of the Faculty of Dentistry, San Luis Potosí University, Mexico.

## 2. Literature Search Strategy

This review was based on an electronic database search through Medline/PubMed, Scopus, and Web of Knowledge, which was conducted to obtain relevant papers published in the English and Spanish languages, between June 1999 and June 2015. In order to identify relevant studies, following Medical Subject Headings (MeSH) terms or key words, in different combinations, and with Boolean Operators, we introduced the following: “*glucose-6-phosphate dehydrogenase*”; “*G6PD*”; “*favism*”; “*children*”; “*pediatric patients*”; “*pediatric dentistry*”; “*dental management*”; and “*dental treatment*.”* Search fields*: “All”.* Limits*: “Within the last 15 years”, “Humans”, “English and Spanish”, and “Birth through 18 years”. We also conducted a manual search of relevant papers published in five major international pediatric dentistry journals (Journal of Dentistry for Children, Pediatric Dentistry, The Journal of Clinical Pediatric Dentistry, International Journal of Paediatric Dentistry, and European Journal of Paediatric Dentistry).

Both electronic and manual searching yielded in total 87 articles matching these criteria, and 23 of these were selected for further evaluation. After screening the titles and abstracts of these potentially appropriate articles, plus their reference lists, 10 full-text studies were finally retrieved (one published in 1995) and reviewed by two independent authors (APG and AGR). Relevant information and data were extracted, organized, and summarized for inclusion in this review.

Interestingly, of the 10 articles reviewed (nine in English and one in Spanish), only two were directly related to the field of dentistry, one of which was a pediatric case report about a child with G6PD and Apert syndrome [7]. The clinically relevant data of each report are summarized in Table 1.

## 3. Case Report

In the autumn of 2014, a 9-year-and-2-month-old male child arrived at the Postgraduate Pediatric Dental Clinic at the Faculty of Dentistry, San Luis Potosí University, Mexico, referred by a general medical practitioner, with a chief complaint of “*prolonged retention of upper central primary incisors*.” His parents (aged 55 and 34 years, resp.) and female sibling were, in general, healthy. The parents did not report any background of craniofacial trauma, drug or environmental allergies, or previous surgeries. The child was from the mother's second pregnancy and was born at the expected term date, by vaginal delivery, with no natal or perinatal complications; his immunization scheme was complete. According to his hospital clinical history, at the age of 1 year and 4 months, the child was diagnosed with glucose-6-phosphate dehydrogenase (G6PD) deficiency when he suffered a hemolysis crisis with severe anemia after ingesting fava beans; at that time, child's hemoglobin count was 4 g/dL (normal value: 11 g/dL); therefore, the child was administered packed red blood cells (RBC). A complete medical evaluation and several hematology tests, one of these consisting of the measurement of RBC enzyme activity through quantitative spectrophotometry, confirmed the diagnosis. The parents reported another, but moderate, hemolysis crisis, which had taken place in August 2014, which required the administration of folic acid and iron.

When the child was physically examined, he displayed a good general health and asymptomatic status; initially, the child exhibited uncooperative behavior, without evidence of psychological disturbances.

This was his first visit to the pediatric dentist. The patient's oral cavity exhibited nearly completely erupted mixed dentition, with severe incisor crowding in maxilla and mandible, moderate overjet (5 mm), and poor hygiene with fair plaque accumulation; none of the teeth demonstrated a beyond-normal mobility, except for the persistent primary upper central incisors. Soft tissue did not exhibit any abnormality. Deep carious cavities were present in both lower primary first molars and in addition to swallow cavities in the four primary second molars and four first permanent molars. The parents mentioned moderate dental-pain episodes in the child, especially during eating.

According to the information collected, interconsultation with the pediatric hematologist, and considering that the patient was systemically stable, we decided to carry out whole oral rehabilitation under local anesthesia. Then parents understood this fully and agreed to the planned treatment by means of signed, written informed consent. The treatment consisted of extractions, pulp therapy, composite restorations, and a space maintainer. In addition, the child and his parents received clear instructions regarding oral hygiene and nutritional advice. Future control reviews were carefully scheduled.

## 4. Discussion

G6PD deficiency was first discovered in 1956 by Carson et al. [14] according to our search of the literature. Since then, a few reports have been published in the dentistry field, with only one concerning a pediatric patient [7]. Although G6PD deficiency or “favism” is a very rare disease, pediatric dentists should be able to recognize this condition and, additionally, its current systemic conditions of the affected child when planning the management of these patients. Clinicians should as well consider that, typically, this disorder exhibits no overt and distinctive physical, facial, or bucodental signs; so a thorough and careful clinical and family history is particularly important, and several critical items may give a clue or reveal an underlying metabolic disorder; furthermore, some parents may be unaware of the condition in their children [2].

It is also necessary to take into account that some uncontrolled oral infectious processes or some drugs commonly used or prescribed in dentistry particularly in children, such as Prilocaine, topical Benzocaine, Acetaminophen, Aspirin, Midazolam, Penicillin (and other antimicrobial agents such as sulphonamides), and certain general anesthetic agents, may induce hemolysis (methahemoglobinemia), with some potential, but exceptionally rare, serious consequences to the subject's general health, including severe anemia, acute renal failure, or malignant hyperthermia [3, 8, 15]. Inhalation sedation is usually safe, and, when indicated, general anesthesia must be administered in a hospital environment [2]. In this regard and according to Elyassi and Rowshan [3], infection is probably the most common factor inciting hemolysis in G6PD-deficient children; a variety of diverse pathogenic microbial agents have been noted, including* Escherichia coli*,* Rickettsia*, pneumonia,* Cytomegalovirus*, viral A and B hepatitis, typhoid fever, beta-hemolytic streptococci [11], and, quite importantly, those related to deep carious lesions and maxillofacial infections [10, 12]. It is also noteworthy that the severity of the hemolysis depends on several factors, including concomitant drug administration, liver function, and the patient's age [11].

Methahemoglobinemia is a conversion of defective iron existing in hemoglobin to a ferric form that does not allow such good availability of oxygen to the tissues, which can result in cyanosis [2]. When a hemolysis crisis occurs in a child with G6PD deficiency after taking a potential drug, receiving a general anesthetic agent or ingesting fava beans, the first clinical manifestations (cyanosis, headache, dyspnea, paleness, hemoglobinuria, fatigue, lumbar/substernal pain, jaundice, scleral icterus, and dark urine) arise within 24–72 h, and acute anemia worsens until about day 7. Therefore, it is important to maintain a close postoperative follow-up in order to check the patient's health. In the case of the occurrence of a hemolysis crisis, there is no specific treatment, but the offending drug must be discontinued; it has been mentioned that hemoglobin concentrations begin to recover after 8–10 days [3, 11]. The need for blood transfusions in children is rarely indicated and is only in the most severe cases, for example, when the affected patient goes into shock. Mainly the pediatric hematologist should determine this therapeutic action.

## 5. Conclusions

It is necessary for pediatric dentists to know how to recognize children with suspected G6PD or favism, not only for counseling the parents, but also for taking all precautions regarding dental restorative treatment, particularly when general anesthesia is indicated. The best management strategy to prevent adverse consequences in these patients is to avoid delivering any kind of oxidative stressors; in cases of painful and anxious children, it is mandatory to provide only appropriate drugs, which are safe and have not been shown to cause hemolytic crisis. Further, rigorous bucodental preventive management is always strongly recommended.

## Figures and Tables

**Table 1:** Full-text article finally retrieved and reviewed.

Author(s)	Article type/main findings reported
Chang and Liu [5]	General review/“It is widely accepted that the high frequency of G6PD deficiency has evolved because of the selective pressure exerted by *Plasmodium falciparum *malaria.”

Mehta et al. [9]	General review/“… there is a risk of neonatal jaundice and acute haemolytic anaemia, triggered by infection and the ingestion of certain drugs and broad beans (favism).”

Quereshy et al. [10]	Dental case report of a 30-year-old African American man/G6PD as a result of a local maxillofacial infection subsequent to a mandibular tooth extraction.

Frank [6]	Diagnosis and management review/the diagnosis of G6PD deficiency is made by a quantitative spectrophotometric analysis or, more commonly, by a rapid fluorescent SPOT test.

Tosun and Sener [7]	Dental case report of a 4-year-old male with Apert syndrome and favism. The patient was in the long term followed up/“… therefore, professional care is necessary and care must be taken for either drug-induced haemolysis or infection, as a result of G6PD deficiency.”

Cappellini and Fiorelli [11]	General review (seminar)/“Fortunately, most G6PD-deficient individuals are asymptomatic throughout their life, and unaware of their status.”

Elyassi and Rowshan [3]	Literature review on the perioperative management of G6PD in anesthesiology/“… management of pain and anxiety should include medications that are safe and have not been shown to cause hemolytic crisis, such as benzodiazepines, Codeine/Codeine derivatives, Propofol, Fentanyl, and Ketamine.”

Youngster et al. [8]	An evidence-based review on medications and G6PD/“… we found solid evidence to prohibit only seven currently used medications: Dapsone, methylthioninium chloride (Methylene Blue), Nitrofurantoin, Phenazopyridine, Primaquine, Rasburicase, and tolonium chloride (Methylene Blue).”

Verdugo et al. [12]	Case report of a Chilean 2-year-and-7-month-old boy/“Among the most common clinical manifestations of this condition, acute hemolysis, chronic hemolysis, neonatal hyperbilirubinemia, and an asymptomatic form are observed.”

Monteiro et al. [4]	Systematic review on prevalence in Latin America/“Low prevalence rates of G6PDd were documented in Argentina, Bolivia, Mexico, Peru, and Uruguay, but studies from Curaçao, Ecuador, Jamaica, Saint Lucia, and Trinidad, as well as some surveys carried out in areas of Brazil, Colombia, and Cuba, have shown a high prevalence (>10%).”
